# Overexpression of Interferon-Inducible Protein 16 Promotes Progression of Human Pancreatic Adenocarcinoma Through Interleukin-1β-Induced Tumor-Associated Macrophage Infiltration in the Tumor Microenvironment

**DOI:** 10.3389/fcell.2021.640786

**Published:** 2021-06-04

**Authors:** Jing-Xian Chen, Chien-Shan Cheng, Hong-Fang Gao, Zi-Jie Chen, Ling-Ling Lv, Jia-Yue Xu, Xiao-Heng Shen, Jing Xie, Lan Zheng

**Affiliations:** ^1^Department of Traditional Chinese Medicine, Shanghai Jiao Tong University School of Medicine Affiliated Ruijin Hospital, Shanghai, China; ^2^Department of Integrative Oncology, Fudan University Shanghai Cancer Center, Shanghai, China; ^3^Department of Oncology, Shanghai Medical College, Fudan University, Shanghai, China; ^4^Department of Oncology, Shanghai Yangpu Hospital of Traditional Chinese Medicine, Shanghai, China; ^5^Department of Geriatrics, Shanghai Yangpu Hospital of Traditional Chinese Medicine, Shanghai, China

**Keywords:** IFI16, inflammasome, pancreatic adenocarcinoma, tumor-associated macrophages, IL-1β

## Abstract

Activation of inflammasomes has been reported in human pancreatic adenocarcinoma (PAAD); however, the expression pattern and functional role of inflammasome-related proteins in PAAD have yet to be identified. In this study, we systemically examined the expression and role of different inflammasome proteins by retrieving human expression data. Several genes were found to be differentially expressed; however, only interferon-inducible protein 16 (IFI16) expression was found to be adversely correlated with the overall survival of PAAD patients. Overexpression of IFI16 significantly promoted tumor growth, increased tumor size and weight in the experimental PAAD model of mice, and specifically increased the population of tumor-associated macrophages (TAMs) in the tumor microenvironment. Depletion of TAMs by injection of liposome clodronate attenuated the IFI16 overexpression-induced tumor growth in PAAD. *In vitro* treatment of conditioned medium from IFI16-overexpressing PAAD cells induced maturation, proliferation, and migration of bone marrow-derived monocytes, suggesting that IFI16 overexpression resulted in cytokine secretion that favored the TAM population. Further analysis suggested that IFI16 overexpression activated inflammasomes, thereby increasing the release of IL-1β. Neutralization of IL-1β attenuated TAM maturation, proliferation, and migration induced by the conditioned medium from IFI16-overexpressing PAAD cells. Additionally, knockdown of IFI16 could significantly potentiate gemcitabine treatment in PAAD, which may be associated with the reduced infiltration of TAMs in the tumor microenvironment. The findings of our study shed light on the role of IFI16 as a potential therapeutic target for PAAD.

## Introduction

Pancreatic cancer, mainly in the form of pancreatic adenocarcinoma (PAAD), is one of the most malignant human cancers worldwide. PAAD is the fourth common cause of cancer-related death currently and has been projected to be the second one, after lung cancer, by 2030 (Rahib et al., 2014). Despite the rapid development in diagnostic technology and new treatments, PAAD is still very difficult to detect at an early stage, which results in a delayed intervention that largely causes poor prognosis in the patients (Oberstein and Olive, 2013). Surgical resection is the main optimal treatment; however, in patients with non-surgical PAAD, chemotherapeutic agents, such as gemcitabine, demonstrate a very poor response (Principe et al., 2020). The identification of novel diagnostic and therapeutic targets is necessary. Inflammation plays an important role in the pathogenesis of PAAD (Stone and Beatty, 2019). Patients with chronic pancreatitis are at a high risk of developing PAAD (Yadav and Lowenfels, 2013), and an experimental model of pancreatitis could reportedly accelerate PAAD progression (Carriere et al., 2009). At the cellular level, it was clinically observed that pro-inflammatory immune cells, such as macrophages, accumulate in PAAD (Deschenes-Simard et al., 2013). Release and infiltration of pro-inflammatory cytokines, as well as activation of pro-inflammatory signaling, including NF-κB, COX-2, and TLRs, also demonstrate the involvement of inflammation in PAAD (Pramanik et al., 2018). Thus, inflammation may be a potential target for the discovery of a new therapeutic strategy for PAAD.

The inflammasome is a cellular protein complex that mediates the inflammatory response toward various pathogenic microorganisms and sterile sensors (Guo et al., 2015). Particularly, the activation of inflammasomes in sterile inflammation was found to play an important role in the pathogenesis and progression of human cancers, including PAAD (Xu et al., 2019). Activation of the AIM2 inflammasome during the pathogenesis of PAAD caused HMGB1 release, which conferred an immunosuppressive tumor microenvironment and led to tumor cell immune evasion (Li et al., 2018). The product of inflammasome activation, IL-1β, was significantly increased in pancreatic cancer tissue. Inhibition of inflammasome activation prevents infiltration of IL-1β that retards pancreatic cancer cell proliferation (Mohammed et al., 2017). Inflammasome activation in PAAD tumor cells may also regulate stromal cells, such as cancer-associated fibroblasts, through IL-1β/IL-1R (Brunetto et al., 2019). Additionally, the activation of platelet inflammasomes in PAAD was found to positively regulate platelet aggregation and tumor growth in mice (Boone et al., 2019). These lines of reports indicate that targeting the inflammasome may be a potential therapeutic target for the treatment of PAAD. However, a systematic investigation of the expression profile of inflammasome-related molecules and their role in PAAD remains lacking.

Interferon-inducible protein 16 (IFI16) is a HIN-200 protein which contains a 200-amino-acid DNA binding domain at its C-terminus and a PYRIN domain at its N-terminus (Liao et al., 2011). As a DNA sensor of inflammation, IFI16 plays a critical role in the regulation of gene transcription and cellular response to stress (Choubey and Panchanathan, 2016). IFI16 regulates the inflammation response of cells in various mechanisms. In viral infection, IFI16 binds to viral double-stranded DNA (dsDNA) to activate STING-TBK1 for the production of IFN-β (Unterholzner et al., 2010). At the same time, the binding of IFI16 with ASC and procaspase-1 may form a DNA inflammasome complex that triggers IL-1β mutation and release (Zhao et al., 2015). In human cancers, the role of IFI16 may be far from conclusive. It was even found that IFI16 expression was reduced in liver cancer and may act as a tumor suppressor gene by triggering cell apoptosis and inhibiting cell proliferation (Lin et al., 2017). However, a controversial argument of the role of IFI16 in cancer was found in both oral cancer and renal clear cell carcinoma, in which IFI16 may serve as an oncogene to promote cell proliferation and tumor progression (Kondo et al., 2012; Yu et al., 2021). The role of IFI16 in mediating change in the tumor microenvironment remains unknown.

In this study, we profiled the expression pattern of inflammasome-related proteins in human PAAD by retrieving data from publicly available human cancer databases and identified its correlation with the survival of human cancer patients. Molecules that are differentially expressed and correlated with the survival of PAAD patients were studied to elucidate their functional role in tumor growth and immune response in the tumor microenvironment. Furthermore, we investigated the mechanism through which inflammasome-related proteins in the PAAD cells mediate the tumor microenvironment-driven development and progression of PAAD.

## Materials and Methods

### Chemical, Plasmids, and Antibodies

Liposome PBS and liposome clodronate were obtained from Liposoma BV (Netherlands). Gemcitabine, bromodeoxyuridine (BrdU), and PKH26PCL were purchased from Sigma-Aldrich (United States). Calcein AM was purchased from Thermo Fisher (United States). Poly dA:dT and neutralizing antibody against IL-1β were obtained from InvivoGen (United States). The CRISPR-cas activation plasmid and IFI16 shRNA were purchased from Santa Cruz (United States). Antibodies against IFI16, caspase-1, AIM2, IL-1β, ASC, and β-actin were purchased from Abcam (Cambridge, United Kingdom). FITC-conjugated antibodies against F4/80, APC-conjugated antibodies against CD11b, PE/Cy7-conjugated antibodies against CD11c, APC-conjugated antibodies against CD3, FITC-conjugated antibodies against CD4, and PE/Cy7-conjugated antibodies against CD8 were purchased from BioLegend (United Kingdom).

### Cell and Cell Culture

The murine PAAD cell line, Panc-2, was obtained from the Frederick National Laboratory for Cancer Research (Frederick, MD, United States) and has been used in our previous study (Gao et al., 2019). Panc-1, BxPC3, and SW1990 cell lines were obtained from the American Type Culture Collection (ATCC, United States). All cells were cultured in DMEM, supplemented with 10% FBS and 1% penicillin/streptomycin (Thermo Fisher, United States), under humidified conditions of 37°C and 5% CO_2_.

### Orthotopic PAAD Murine Model

The orthotopic PAAD murine model was established according to the protocol described in our previous study (Gao et al., 2020). The animal study protocol was approved by the Animal Experimental Ethics Committee of Ruijin Hospital, Shanghai Jiao Tong University School of Medicine. Briefly, luciferase-tagged Panc-2 cells were mixed with the Matrigel matrix (BD Bioscience, United States). A 20-μl mixture containing 1 × 10^8^ Panc-2 cells was injected into the pancreas of C57BL/J mice. Measurement of orthotopic tumor size was performed once per week, beginning 1 week post injection, using the IVIS Spectrum live animal imager (PerkinElmer, United States), with luciferin (30 mg/kg, i.p.) as the substrate. For the gemcitabine treatment, mice were orally administered gemcitabine at a dose of 100 mg/kg daily. At the end of the study, the mice were sacrificed, and the pancreas was dissected out.

### Tumor-Associated Macrophage Depletion by Liposome Clodronate

Depletion of Tumor-Associated Macrophages (TAMs) was performed according to a published protocol, with minor modifications (Jordan et al., 2003). To minimize the early recruitment of TAMs, mice received a single injection of liposome PBS (as sham control) or liposome clodronate 3 days before orthotopic implantation of tumor cells and then subsequent injections twice per week after implantation at a dose of 15 mg/kg.

### Isolation and Culture of Bone Marrow-Derived Macrophages

Bone Marrow-Derived Macrophages (BMDMs) were isolated using the Ficoll method, according to a published protocol (Pinton et al., 2019). Briefly, the femurs of C57BL/J mice were isolated, and monocytes were flushed out. Monocytes were enriched by gradient centrifugation using the Ficoll reagent. The enriched monocytes were then cultured in RPMI culture medium, supplemented with 10% FBS and 10 ng/ml recombinant murine M-CSF, for 7 days.

### BrdU Incorporation Assay

For the *in vitro* BrdU incorporation assay, 10 μM BrdU was added to the culture medium 4 h before sample collection by trypsinization. For the *in vivo* BrdU incorporation assay, 10 mg/kg BrdU was intraperitoneally injected into the mice 24 h before sample collection, by enriching TAMs from the dissected tumor using the Ficoll method. The *in vitro* or *in vivo* collected cells were then processed according to the manufacturer’s instructions (Promega, United States). Briefly, collected cells were stained with appropriate cell surface markers. The cells were then fixed and penetrated with fixatives for 2 h at room temperature, followed by incubation with 1 μg/mL FITC-conjugated anti-BrdU antibody for 30 min. The cells were then subjected to flow cytometry analysis.

### Transwell Cell Migration Assay

Quantitative analysis of BMDM migration was performed using the Transwell cell migration assay. Briefly, 2 × 10^5^ BMDMs cultured in conditioned medium from Panc-2 cells were seeded at the apical side of Transwell inserts with serum-free medium. Culture medium, supplemented with chemotaxis MCP-1 (10 ng/ml), was added to the receiving chambers and cultured for 3 h. Cells remaining on the apical side of the inserts were scraped away, and cells at the basal side of the membrane were collected by trypsinization. The cells were then collected and stained with 50 μM calcein AM and quantified using a fluorescence microplate reader (PerkinElmer, Germany).

### Co-culture System

The Panc-2 cells were cultured on the apical side of the 0.8-μM-pore size Transwell, while BMDMs were seeded on the receiving chamber with the non-attached surface, supplemented with 10 ng/ml M-CSF. This co-culture was maintained for 7 days to allow all possible non-contact interactions between the Panc-2 cells and BMDMs. BMDMs were then collected for analysis of the TAM population with flow cytometry and quantitative real-time PCR (qPCR).

### Blocking IL-1β With Neutralizing Antibody

Supplementation with IL-1β-neutralizing antibody mitigated the function of secreted IL-1β in the conditional medium from Panc-2 cells. This method was adopted from a published protocol, with minor modifications (Staudt et al., 2013). For the blocking experiments, the BMDMs were preincubated with 10 μg/ml of IL-1β-neutralizing antibody for 15 min before the functional studies.

### Flow Cytometry

Cells were stained with various fluorescence-conjugated antibodies for 15 min in the dark at room temperature. Cells (1 × 10^6^) were stained with 1 μg of antibody and washed with 300 μl of PBS. The cells were then resuspended in 300 μl of PBS for analysis with flow cytometry (Canto II, BD Bioscience, United States). To sort TAMs, dissected tumors were digested with 0.8 mg/ml of collagenase IV for 30 min at 37°C with gentle shaking. TAMs were enriched using the Ficoll method and then stained with the cell surface markers F4/80 and CD11b. The F4/80 + CD11b + TAMs were then collected using a cell sorter (Aria I, BD Bioscience, United States).

### Quantitative Reverse-Transcription PCR

Total RNA was extracted using the TRIzol method (Life Technologies, United States). First-strand cDNA was prepared using a reverse-transcription kit (Life Technologies, United States). SYBR Green qRT-PCR was performed to measure the gene expression on a qPCR platform (Bio-Rad, United States) with specific primer pairs as follows: HIF-1α (forward: 5′-TGATGTGGGTGCTGGTGTC-3′, reverse: 5′-TTGTGTTGG GGCAGTACTG-3′), CCL2 (forward: 5′-AGGTCCCTGTCAT GCTTCTGG-3′, reverse: 5′-CTGCTGCTGGTGATCCTCTTG- 3′), PECAM1 (forward: 5′-CCAAAGCCAGTAGCATCATGG TC-3′, reverse: 5′-GGATGGTGAAGTTGGCTACAGG-3′), IF N-γ (forward: 5′-CAGCAACAGCAAGGCGAAAAAGG-3′, re verse: 5′-TTTCCGCTTCCTGAGGCTGGATα-3′), TGF-β (for ward: 5′-ACTGATACGCCTGAGTGGCT-3′, reverse: 5′-CCCT GTATTCCGTCTCCTTG-3′), and β-actin (forward: 5′-AAGG CCAACCGTGAAAAGAT-3′, reverse: 5′-GTGGTACGACCAG AGGCATAC-3′) as control.

### Immunoblotting

Total protein was isolated by gel electrophoresis and transferred onto a polyvinylidene fluoride (PVDF) membrane (Millipore, United States). The membrane was then blocked using 10% BSA in TBST buffer at room temperature, for 2 h, and then incubated with primary antibodies overnight at 4°C. After washing, the membrane was incubated with the appropriate secondary antibodies at room temperature for 2 h. The bands were then read by ChemiDoc chemiluminescence using ECL Select as the substrate (Bio-Rad, United States).

### Bioinformatic Analysis

Expression data of related genes in human pancreatic cancer were extracted from GEPIA^1^ (Tang et al., 2017). GEPIA data were collected from the GCGA and GTEx projects using a standard processing pipeline. For the selection of inflammasome-related genes, we selected the key proteins shortlisted from GeneCards-listed inflammasome-related genes by including only those genes encoding intracellular proteins that are composed of inflammasome machinery. Data are presented as dot blots of transcripts per million in the sequencing. Gene expressions with a fold change of over 2 and a *p*-value lower than 0.05 were considered statistically significant in the difference between normal and tumor tissues. The Kaplan–Meier plot was automatically generated by GEPIA using the median expression of a particular gene as the group cutoff. For the correlation analysis, data of particular gene pairs were extracted from GEPIA and analyzed using Spearman’s correlation coefficient. The results used a non-log scale for calculation and the log-scale axis for visualization. Two datasets, GDS4336 and GDS4103, were collected from the GEO^2^ of NCBI and were analyzed by paired Student’s *t*-test, with *p* < 0.05 considered statistically significant between groups.

### Statistical Analysis

Experiments were performed in triplicate. Data are presented as mean ± SEM. Statistical analysis was performed using Student’s *t*-test. Differences were considered statistically significant at *p* < 0.05.

## Results

### Expression of IFI16 Was Increased in PAAD and Correlated to Poor Patient Prognosis

**FIGURE 1:**
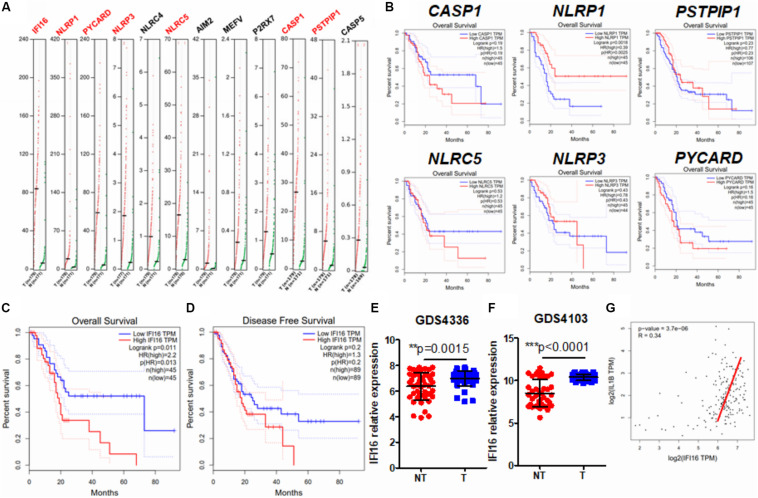
IFI16 was overexpressed in human PAAD and correlated with poor survival of the patients. **(A)** Expression data of inflammasome-related proteins were retrieved from the human database GEPIA. A comparison of expression between normal pancreatic tissues and PAAD tissues was done. Genes with a significantly different expression between normal and tumor tissues are shown in red. **(B)** The data on overall survival and disease-free survival of PAAD patients were retrieved. Only NLRP1, among the genes with significantly different expression, showed a negative correlation with patients’ overall survival. Overall survival **(C)** but not disease-free survival **(D)** was adversely correlated with the expression of IFI16 in PAAD. We further extracted expression data of IFI16 from the human GEO database, including GDS4336 **(E)** and GDS4103 **(F)**. IFI16 was significantly overexpressed in the tumor tissues of PAAD compared with that in the non-tumor adjacent normal pancreas. **(G)** Pearson correlation between the expression of IFI16 and IL-1β was analyzed, which showed a positive correlation in human PAAD. *p < 0.05.

Several studies have observed inflammasome activation during tumorigenesis and progression of PAAD (Brunetto et al., 2019; Yaw et al., 2020); however, the expression pattern of inflammasome-related genes and their functional roles have yet to be systematically investigated. To profile the expression pattern, we extracted human expression profiles of inflammasome-associated genes in PAAD from the GEPIA database (Figure 1A). We selected the genes according to a literature review and GeneCards viewing of the machinery proteins involved in inflammasome priming. Both classic and non-classic pathways of inflammasome priming were searched, but only those proteins that directly compose the inflammasome complex were included, as we were not able to include all other proteins that indirectly regulate inflammasome pathways, owing to the size of the study. Only intracellular proteins were included; therefore, secreted proteins, such as IL-1β and IL-18, were ruled out. Seven of 12 inflammasome-related genes, namely, IFI16, NLRP1, PICARD, NLRP3, NLRC5, CASP1, and PSTPIP1, were found to be upregulated in PAAD compared to those in normal pancreatic tissues. To understand the clinical significance of the upregulation of genes, we extracted the data of patients’ overall survival and plotted the survival curves of patients grouped by median expression of the individual gene (Figure 1B). A significant difference in overall survival was observed in patients grouped by median expression of NLRP1 and IFI16; however, a high expression of NLRP1 predicted better survival of PAAD patients. The two results are not in line with the function of NLRP1 in PAAD; therefore, we excluded this gene from further analysis. This contradiction may result from several different factors and may suggest that rather than an initiating factor in PAAD progression, NLRP1 overexpression may act as a response to restrict the growth and expansion of PAAD tumors at a particular stage to a certain level. Interestingly, only a lower expression of IFI16 predicted better patient survival (Figure 1C). IFI16 expression was not significantly correlated with the disease-free survival of PAAD patients (Figure 1D), suggesting that IFI16 may not be related to the recurrence of the disease in patients who have received particular treatment such as surgical resection but may mainly be the factor of gross survival of the patients. This means that IFI16 overexpression may not be indicative in the short-term disease period once patients receive treatment but may suggest an unfavorable long-term outcome of survival of PAAD patients. The expression of IFI16 was further examined in two datasets of human PAAD samples, which revealed that IFI16 was significantly overexpressed in tumor tissues than in non-tumor adjacent tissues in both datasets (Figures 1E,F). Since IL-1β production is a common consequence of inflammasome activation, we extracted data on the expression of both IFI16 and IL-1β in PAAD. The analysis showed that the expression of IL-1β was positively correlated with the expression of IFI16, further suggesting the activation of inflammasomes in PAAD (Figure 1G). This observation indicates that IFI16 is overexpressed in PAAD, which correlates with the activation of the inflammasome and poor survival of patients.

### Overexpression of IFI16 Accelerates Orthotopic Growth of PAAD in Mice

**FIGURE 2:**
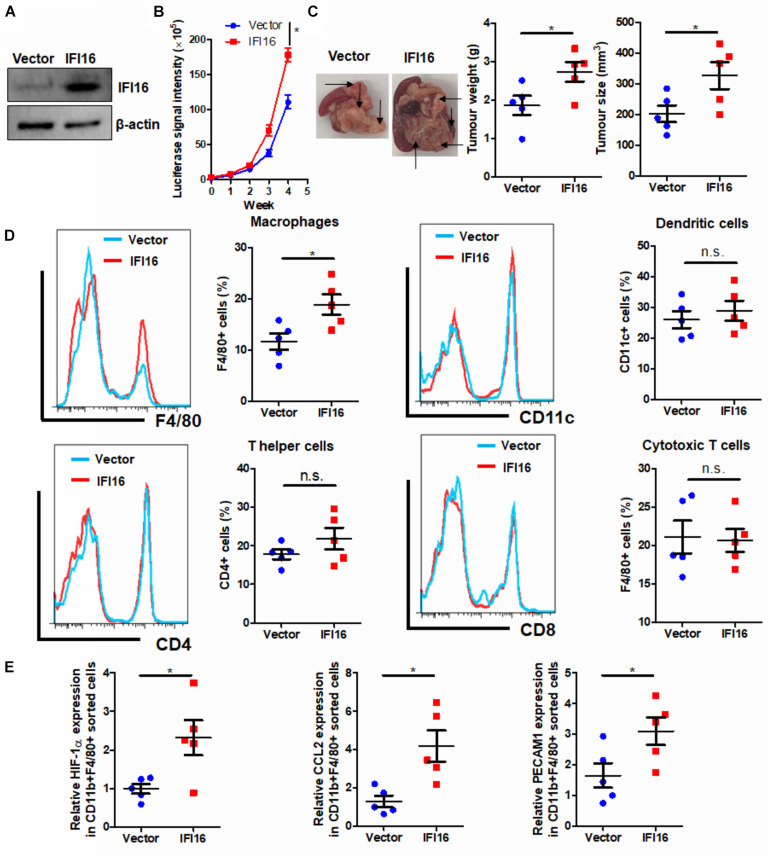
Overexpression of IFI16 promoted tumor growth in an experimental model of PAAD. **(A)** The IFI16-overexpressing PAAD cell line was established by transfecting the CRISPR activation plasmid of IFI16 into the Panc-2 cells. Cells were selected with a culture medium containing 1 μg/ml of puromycin, until a stable IFI16-overexpressing clone was established. Overexpression of IFI16 was validated with immunoblotting. **(B)** Panc-2 of a stable clone expressing the luciferase reporter was established. Panc-2 cells were transfected with the PGL3 vector expressing firefly luciferase and selected by neomycin (50 μg/ml). Approximately 20 μg Matrigel matrix, containing 1 × 10^8^ Panc-2 cells, was orthotopically injected into the pancreas of mice. The luciferase signal intensity was measured by intraperitoneal injection of luciferin (30 mg/kg) and quantification under a live animal imager once per week. Overexpression of IFI16 significantly accelerated the orthotopic growth of the pancreatic tumor. **(C)** At the end of the study, the mice were sacrificed, and the pancreas along with the spleen was dissected out. The tumor weight was measured, and tumor size was calibrated by the diameters of the tumor. Overexpression of IFI16 potently increased the tumor size and weight in the experimental PAAD model. The black arrow shows an obvious surface tumor nodule found in the pancreas. **(D)** The dissected tumor was then digested in 0.8 mg/ml of collagenase IV for 30 min at 37°C with gentle shaking. The immune cells were enriched with Ficoll methods. The profile of immune cells in the tumor microenvironment was measured with flow cytometry. Overexpression of IFI16 significantly increased the population of TAMs but not dendritic cells, T helper cells, or cytotoxic T cells. **(E)** The F4/80 + CD11b + TAMs were collected using a cell sorter, and expressions of HIF-1α, CCL2, and PECAM1 were measured with qRT-PCR. TAMs from IFI16-overexpressing tumors exhibited a significantly high expression of TAM markers, including HIF-1α, CCL2, and PECAM1. *p < 0.05.

IFI16 is an intracellular protein that responds to DNA damage during the initiation of inflammasome activation (Choubey and Panchanathan, 2016). To identify the functional role of IFI16 in mediating PAAD progression, we stably overexpressed this protein in Panc-2 cells (Figure 2A). The wild-type and IFI16-overexpressing Panc-2 cells were orthotopically injected into the pancreas of mice, and the formation and growth of orthotopic tumors were observed by weekly measurement of the luciferase activity for 4 weeks. Overexpression of IFI16 in Panc-2 cells significantly accelerated tumor growth by a week (Figure 2B). At the end of the experiment, tumors were dissected out; representative tumors in each group are shown in Figure 2C. The weight of the tumor was then measured, which indicated that IFI16 overexpression remarkably increased the tumor weight (Figure 2C). Since IFI16 overexpression was reported to trigger innate immune cells (Unterholzner et al., 2010), we measured whether IFI16 overexpression in PAAD altered the immune cell profile in the tumor microenvironment. PAAD tissues with or without IFI16 overexpression were digested and were analyzed with flow cytometry using specific marker staining of different immune cells. Interestingly, IFI16 overexpression specifically increased the population of CD11b + F4/80 + macrophages in the tumor microenvironment but had minimal effect on the populations of CD11b + CD11c + dendritic cells, CD3 + CD4 + CD8- T helper cells, and CD3 + CD4-CD8 + cytotoxic T cells, suggesting that the effect of IFI16 overexpression in PAAD cells may specifically target TAMs in the tumor microenvironment (Figure 2D, gating shown in Supplementary Figure 1). To confirm the increased population as TAMs, we sorted the CD11b + F4/80 + cells from the pancreatic tumors using a FACS sorter and quantified the expression of some TAM markers, such as HIF-1α, CCL2, and PECAM1, with qRT-PCR. It was observed that the IFI16-overexpressing Panc-2 cells induced increased expression of HIF-1α, CCL2, and PECAM1 compared to vector-expressing Panc-2, which was consistent with our flow cytometric observation (Figure 2E). Taken together, these observations suggest that IFI16 overexpression promotes tumor growth and progression of PAAD and may play a role in regulating TAMs in the tumor microenvironment.

### TAMs Mediate the IFI16 Overexpression-Induced PAAD Tumor Growth and Progression

**FIGURE 3:**
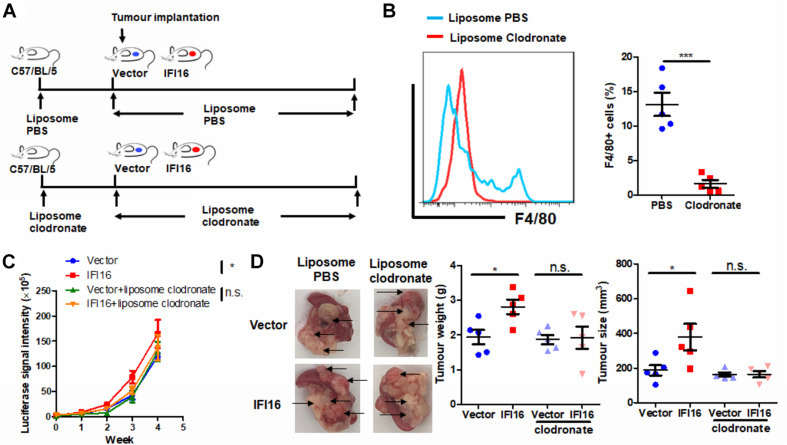
Depletion of TAMs attenuated IFI16-induced tumor growth of PAAD. **(A)** Flowchart of TAM depletion treatment. To minimize early recruitment of TAMs, mice received a single injection of liposome PBS (as sham control) or liposome clodronate, 3 days before orthotopic implantation of tumor cells, and received subsequent injection twice per week after implantation at a dose of 15 mg/kg. **(B)** The tumor was dissected out, and TAMs were enriched with Ficoll methods. The enriched cells were stained with an antibody against F4/80 and subjected to flow cytometric analysis. Treatment with liposome clodronate could potently remove TAMs from mice with orthotopic PAAD tumors. **(C)** Panc-2 of a stable clone expressing the luciferase reporter was established. Panc-2 cells were transfected with the PGL3 vector expressing firefly luciferase and selected by neomycin (50 μg/ml). Approximately 20 μl Matrigel matrix, containing 1 × 10^8^ Panc-2 cells, was orthotopically injected into the pancreas of mice. The luciferase signal intensity was measured by intraperitoneal injection of luciferin (30 mg/kg) and quantification under a live animal imager once per week. Depletion of TAMs attenuated the tumor growth of PAAD induced by IFI16 overexpression. **(D)** At the end of the study, the mice were sacrificed, and the pancreas along with the spleen was dissected out. The tumor weight was measured, and tumor size was calibrated by the diameters of the tumor. Depletion of TAMs abolished the IFI16 overexpression-induced increase in tumor size and weight in the experimental PAAD model. The black arrow shows an obvious surface tumor nodule found on the pancreas. *p < 0.05 and ***p < 0.001.

To further identify the role of TAMs in IFI16 overexpression-induced PAAD tumor growth and progression, we used liposome clodronate to deplete TAMs from mice bearing orthotopic PAAD (Jordan et al., 2003). To minimize the early recruitment of TAMs, mice received a single injection of liposome PBS (as control) or liposome clodronate 3 days before orthotopic implantation of tumor cells and then received subsequent injections twice per week after implantation (Figure 3A). It was observed that injection of liposome clodronate completely removed the TAMs from the tumor microenvironment, as evidenced by the rare appearance of F4/80 + cells in the tumor (Figure 3B, gating shown in Supplementary Figure 2). Injection of liposome PBS had minimal effect on the IFI16 overexpression-induced PAAD tumor growth and progression, suggesting that no vehicle effect was observed. In contrast, injection of liposomal clodronate to remove TAMs significantly abolished the promotion of PAAD growth and progression rate induced by IFI16 overexpression (Figure 3C). The size, as well as weight, of the representative tumor also suggested that IFI16 overexpression could be neutralized upon TAM clearance (Figure 3D). This observation indicates that TAMs are necessary for mediating the promoting effect of IFI16 overexpression on PAAD progression and growth.

### IFI16 Overexpression in PAAD Cells Induces Maturation, Infiltration, and Proliferation of TAMs in the Tumor Microenvironment

**FIGURE 4:**
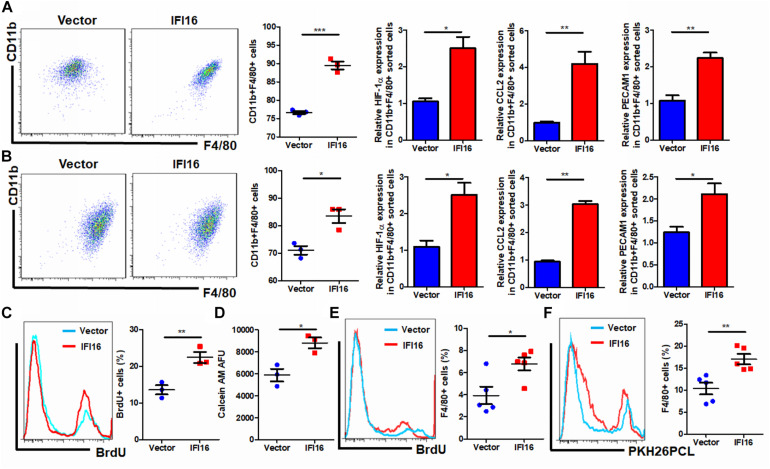
Conditional medium from IFI16-overexpressing PAAD cells increased the TAM population. **(A)** The culture medium of wild-type and IFI16-overexpressing PAAD cells was collected. BMDMs were cultured with 30% of the aforementioned conditional medium for 7 days. Cells were then collected and stained with antibodies against CD11b and F4/80 and subjected to flow cytometric analysis. BMDMs cultured with IFI16-overexpressing PAAD cells showed a higher level of CD11b + F4/80 + cells. Total RNA was extracted, and the expressions of HIF-1α, CCL2, and PECAM1 were analyzed with qRT-PCR. BMDMs cultured with conditional medium from IFI16-overexpressing PAAD cells showed significantly higher expressions of HIF-1α, CCL2, and PECAM1. **(B)** The Panc-2 cells with or without IFI16 overexpression were cultured on the apical side of the 0.8-μM-pore size Transwell, while BMDMs were seeded on the receiving chamber with the non-attached surface, supplemented with 10 ng/ml of M-CSF. This co-culture was maintained for 7 days to allow all possible non-contact interaction between the Panc-2 cells and BMDMs. BMDMs were then collected for analysis of the TAM population with flow cytometry and qPCR. Co-culture of Panc-2 cells with IFI16 overexpression significantly increased the population of TAMs from the BMDM culture compared to the co-culture of vector-expressing Panc-2 cells. **(C)** Added to the culture medium was 10 μM of BrdU 4 h prior to sample collection by trypsinization. BrdU-incorporated CD11b + F4/80 + cells were stained with anti-BrdU antibody and detected with a flow cytometer. BMDMs cultured with the conditional medium from IFI16-overexpressing PAAD cells showed significantly higher incorporation of BrdU into the DNA. **(D)** Approximately 2 × 10^5^ BMDMs cultured with the conditional medium from Panc-2 cells were seeded at the apical side of the Transwell insert with serum-free medium. A culture medium supplemented with chemotaxis MCP-1 (10 ng/ml) was added into the receiving chambers and cultured for 3 h. Cells remaining at the apical side of inserts were scraped away, and cells at the basal side of the membrane were collected by trypsinization. The cells were then collected and stained with 50 μM calcein AM and quantified with a fluorescence microplate reader. BMDMs cultured with the conditional medium from IFI16-overexpressing PAAD cells showed significantly higher motility. **(E)** Intraperitoneally injected into the mice was 10 mg/kg of BrdU 24 h prior to sample collection by enriching TAMs from the dissected tumor by Ficoll methods. BrdU-incorporated CD11b + F4/80 + cells were stained with an anti-BrdU antibody and detected with a flow cytometer. TAMs from tumors with IFI16 overexpression showed significantly high incorporation into the DNA. **(F)** BMDMs were isolated from the femurs and cultured into macrophages in a medium containing 10 μg/ml of M-CSF for 7 days. Cells were then stained with 100 μM PKH26PCL for labeling. Labeled cells were then intraperitoneally injected into the mice bearing PAAD tumors with or without IFI16 overexpression and allowed circulation for 24 h. The tumor was then dissected out, and the number of PKH26PCL-labeled cells infiltrated into the tumor was measured with a flow cytometer. IFI16-overexpressing tumors showed more cell infiltration into the tumor microenvironment. *p < 0.05, **p < 0.01 and ***p < 0.001.

Regulation of the TAM population in the tumor microenvironment may involve multiple processes, including the infiltration of circulating pro-inflammatory monocytes, maturation of infiltrated monocytes, and proliferation of local TAMs (Yang et al., 2018). To understand how IFI16 overexpression in PAAD cells regulates the TAM population in the tumor microenvironment, we collected the culture supernatant from PAAD cells with or without IFI16 overexpression. BMDMs treated with 30% culture supernatant from PAAD cells with IFI16 overexpression exhibited a higher level of CD11b + F4/80 + population after 7-day incubation, suggesting that IFI16 overexpression in PAAD cells can increase the maturation of TAMs from monocytic cells in the tumor microenvironment (Figure 4A). We further co-cultured the Panc-2 cells with BMDMs in a Transwell system. The Panc-2 cells with or without IFI16 overexpression were cultured on the apical side of the 0.8-μM-pore size Transwell, while BMDMs were seeded on the receiving chamber with the non-attached surface, supplemented with 10 ng/ml of M-CSF. This co-culture was maintained for 7 days to allow all possible non-contact interactions between the Panc-2 cells and BMDMs. BMDMs were then collected for the analysis of the TAM population by flow cytometry and qPCR. It was found that the co-culture of Panc-2 cells with IFI16 overexpression significantly increased the population of TAMs from BMDM cultures compared to the co-culture of vector-expressing Panc-2 cells (Figure 4B). qPCR analysis confirmed that TAM markers, including HIF-1α, CCL2, and PECAM1, were induced in the BMDMs receiving stimulus from Panc-2 cells with IFI16 overexpression. Simultaneously, the culture supernatant from PAAD cells with IFI16 overexpression accelerated the proliferation of TAMs, as evidenced by increased BrdU incorporation into the DNA of macrophages differentiated from BMDMs (Figure 4C, gating shown in Supplementary Figure 3). The culture supernatant from the PAAD cells overexpressing IFI16 could attract BMDMs from the upper chamber of the Transwell insert toward the receiving chambers (Figure 4D). To confirm that this action was comparable *in vivo*, we injected BrdU intraperitoneally into mice with orthotopic PAAD tumors with or without IFI16 overexpression. Mice with IFI16-overexpressing PAAD tumors showed increased BrdU incorporation into CD11b + F4/80 + TAMs in the tumor microenvironment (Figure 4E, gating shown in Supplementary Figure 4). Additionally, IFI16 overexpression in orthotopic tumors could significantly increase the infiltration of PKH26PCL-stained BMDMs injected into the mice, suggesting that IFI16 overexpression could accelerate the migration of monocytic cells into the tumor microenvironment (Figure 4F, gating same as in Figure 4E). This observation suggests that IFI16 overexpression regulates TAMs in the tumor microenvironment by inducing their maturation, infiltration, and local proliferation.

### IL-1β Production Is Responsible for the Induced Migration and Proliferation of TAMs in Tumor Microenvironments of IFI16-Overexpressing PAAD

**FIGURE 5:**
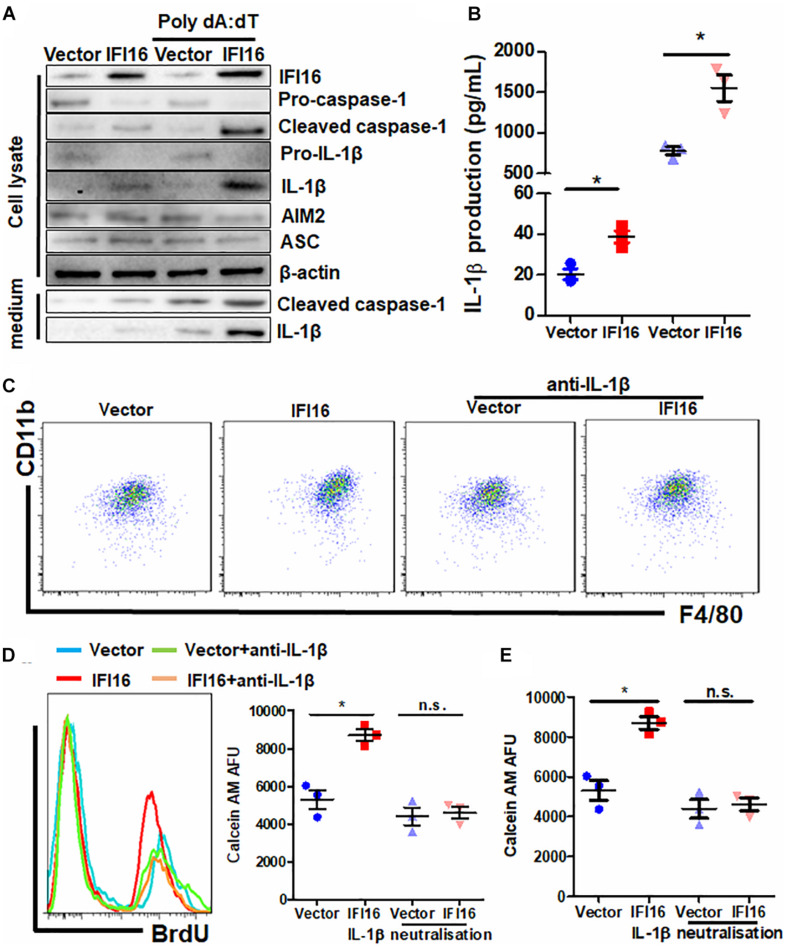
IL-1β is responsible for the IFI16-induced TAM profile changes in PAAD. **(A)** Protein expression of Panc-2 cells at intracellular and extracellular levels was measured with immunoblotting. IFI16 overexpression induced further activation of the inflammasome, as evidenced by the cleavage of intracellular pro-caspase-1 and pro-IL-1β, as well as the extracellular expression of cleaved IL-1β and caspase-1 in the culture medium. **(B)** IL-1β levels were quantified with ELISA. IFI16 overexpression significantly increased IL-1β production and secretion in Panc-2 cells in the presence or absence of poly dA:dT. Supplementation with an IL-1β-neutralizing antibody mitigated the function of secreted IL-1β in the conditional medium from Panc-2 cells. This method was adopted from a published protocol, with minor modifications. For the blocking experiments, the BMDMs were preincubated with 10 μg/ml of the IL-1β-neutralizing antibody, for 15 min before the functional studies. **(C)** Cells were then collected and stained with antibodies against CD11b and F4/80 and subjected to flow cytometry analysis. Neutralization of IL-1β significantly blocked the increase in TAM population cultured in the conditioned medium from IFI16-overexpressing Panc-2 cells. **(D)** Added to the culture medium was 10 μM of BrdU 4 h prior to sample collection by trypsinization. BrdU-incorporated CD11b + F4/80 + cells were stained with anti-BrdU antibody and detected using a flow cytometer. Neutralization of IL-1β significantly blocked the increase in BrdU incorporation into the DNA of BMDMs cultured in conditioned medium from IFI16-overexpressing Panc-2 cells. **(E)** Approximately 2 × 10^5^ BMDMs cultured with the conditioned medium from Panc-2 cells were seeded at the apical side of Transwell inserts with serum-free medium. Culture medium supplemented with chemotaxis MCP-1 (10 ng/ml) was added to the receiving chambers, followed by culture for 3 h. Cells remaining on the apical side of the inserts were scraped away, and cells at the basal side of the membrane were collected by trypsinization. The cells were then collected and stained with 50 μM calcein AM and quantified using a fluorescence microplate reader. Neutralization of IL-1β significantly blocked the increased motility of the BMDMs cultured in the conditioned medium from IFI16-overexpressing Panc-2 cells. *p < 0.05.

IFI16 mediates DNA damage-induced inflammasome activation in cells (Xiao, 2015). To determine whether IFI16 overexpression in PAAD cells induces inflammasome activation, we probed the expression of major proteins related to inflammasomes. Cleavage of pro-caspase-1 and pro-IL-1β was observed in the presence and absence of poly dA:dT, suggesting the initiation of inflammasome machinery (Figure 5A), while other regulatory proteins, such as AIM2 and ASC, remained unchanged. Intracellular and extracellular cleaved forms of caspase-1 and IL-1β, as well as the secretion of mature IL-1β in the culture supernatant, were significantly induced (Figures 5A,B). To further understand how inflammasome activation and IL-1β production by IFI16-overexpressing PAAD cells regulate TAMs, we used neutralizing antibodies to block IL-1β in the culture supernatant from wild-type and IFI16-overexpressing PAAD cells, according to a published protocol with minor modifications (Staudt et al., 2013). The presence of a neutralizing antibody against IL-1β significantly attenuated the increase in the CD11b + F4/80 + population of BMDMs cultured with a supernatant from IFI16-overexpressing PAAD cells (Figure 5C). Similarly, the increased migration and proliferation of TAMs were potently attenuated by the presence of a neutralizing antibody against IL-1β (Figures 5D,E). This observation suggested that the IFI16-induced inflammasome activation in PAAD cells produces IL-1β, which mediates the maturation, migration, and local proliferation of TAMs in the tumor microenvironment.

### Knockdown of IFI16 Suppresses Gemcitabine-Induced TAMs and Increases Its Antitumor Activity

**FIGURE 6:**
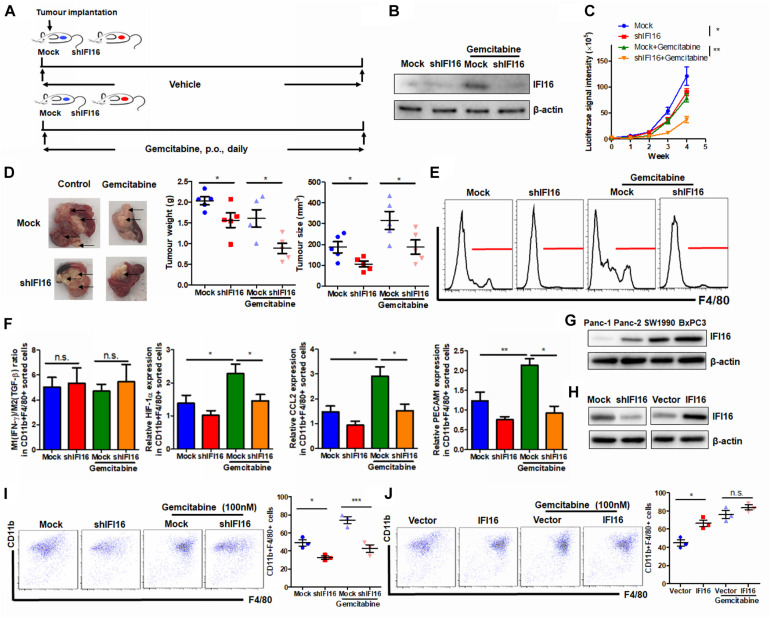
Suppression of IFI16 improves gemcitabine sensitivity in PAAD tumors. **(A)** Flowchart of gemcitabine treatment. Approximately 20 μl Matrigel matrix, containing 1 × 10^8^ Panc-2 cells, was orthotopically injected into the pancreas of mice. Mice were then orally administered gemcitabine at a dose of 100 mg/kg or vehicle every day. **(B)** The IFI16-knockdown PAAD cell line was established by transfecting the shRNA plasmid of IFI16 into the Panc-2 cells. Cells were selected with a culture medium containing 1 μg/ml of puromycin, until a stable IFI16-knockdown clone was established. Knockdown of IFI16 was validated with immunoblotting, which showed that RNA interference using shRNA against IFI16 can significantly attenuate gemcitabine-induced IFI16 upregulation. **(C)** The luciferase signal intensity was measured by intraperitoneal injection of luciferin (30 mg/kg) and quantification under a live animal imager once per week. Knockdown of IFI16 could significantly improve the suppression of tumor growth by gemcitabine. **(D)** At the end of the study, the mice were sacrificed, and the pancreas along with the spleen was dissected out. The tumor weight was measured, and tumor size was calibrated by the diameters of the tumor. Knockdown of IFI16 in gemcitabine-treated mice further reduced the size and weight of the tumor in the experimental PAAD model. The black arrow shows an obvious surface tumor nodule found on the pancreas. **(E)** The tumor was dissected out, and TAMs were enriched by Ficoll methods. The enriched cells were stained with an antibody against F4/80 and subjected to flow cytometry analysis. Knockdown of IFI16 could attenuate the infiltration of TAMs induced by gemcitabine in the tumor microenvironment of PAAD. **(F)** The major M1/M2/TAM markers were quantified using qPCR. The ratio of expression of IFN-γ and TGF-β was calculated to represent the M1/M2 ratio in the tumor microenvironment. Gemcitabine had no significant effect on the polarization of macrophages toward either phenotype but potently activated the expression of TAM markers HIF-1α, CCL2, and PECAM1. **(G)** Expression of IFI16 was measured by immunoblotting in different PAAD cell lines. Expression of IFI16 was highest in BxPC3 cells, then SW1990 cells, and then Panc-2 cells, while Panc-1 cells expressed the lowest level of IFI16. **(H)** Expression of IFI16 was forcefully activated and knocked down in SW1990 cells. **(I)** BMDMs were co-cultured with SW1990 cells with or without IFI16 knockdown in the presence or absence of 100 nM gemcitabine for 7 days. The activation of BMDMs was then examined by flow cytometry. Knockdown of IFI16 reduced co-cultured BMDM activation in the presence or absence of gemcitabine. **(J)** BMDMs were co-cultured with SW1990 cells with or without IFI16 overexpression in the presence or absence of 100 nM gemcitabine for 7 days. The activation of BMDMs was then examined by flow cytometry. Overexpression of IFI16 further increased co-cultured BMDM activation in the presence or absence of gemcitabine. *p < 0.05, **p < 0.01 and ***p < 0.001.

To further examine the regulation of IFI16 in PAAD therapy, we introduced gemcitabine, the major chemotherapeutic agent clinically used for PAAD, to treat mice with orthotopic tumors (Figure 6A). PAAD cells were treated with gemcitabine to induce the expression of IFI16 (Figure 6B). To investigate whether IFI16 plays a role in mediating the sensitivity of PAAD cells in response to gemcitabine treatment, we established a stable IFI16-knockdown clone of PAAD cells (Figure 6B). Knockdown of IFI16 or treatment with gemcitabine moderately reduced the growth rate of orthotopic tumors of PAAD in mice, while the efficacy of gemcitabine in suppressing PAAD tumor was largely improved when IFI16 was knocked down (Figure 6C). Similarly, the size and weight of representative tumors suggested that IFI16-knockdown tumors responded better to gemcitabine treatment than the wild-type tumors (Figure 6D). Interestingly, we observed that gemcitabine treatment could induce TAM population in the tumor microenvironment of orthotopic PAAD and that knockdown of IFI16 could significantly abolish the increase in the TAM population (Figure 6E). To detect the sub-phenotype of the TAMs after gemcitabine treatment, we used qRT-PCR to detect the M1-like macrophage marker IFN-γ and M2-like macrophage marker TGF-β and calculated the ratio of M1/M2 by comparing their expressions. Interestingly, we found that in both mock and IFI16-knockdown tumors, gemcitabine treatment had no significant effect on the ratio of M1/M2 macrophages in the tumors (Figure 6F). This observation is consistent with a previous report stating that gemcitabine had no significant effect on the polarization of macrophages toward either phenotype (Cullis et al., 2017; Kleinerman et al., 2018). However, in our study, when we measured the markers of TAMs, including HIF-1α, CCL2, and PECAM1, we found that gemcitabine treatment potently activated the expression of TAM markers, while knockdown of IFI16 reversed the increase in TAMs in the tumor microenvironment (Figure 6F). To further prove that the effect of IFI16 is not only restricted in Panc-2 cells, we screened the PAAD cell lines in stock in our laboratory, including Panc-1, SW1990, BxPC3, and Panc-2. We found that expression of IFI16 was highest in BxPC3 cells, while lowest in Panc-1 cells, while expression of IFI16 in SW1990 was higher than that in Panc-2 cells (Figure 6G). We then knocked down and overexpressed the IFI16 expression in SW1990 cells (Figure 6H) and co-cultured the cells with BMDMs in the presence or absence of 100 nM of gemcitabine. BMDMs showed less activation into the CD11b + F4/80 + phenotype when co-cultured with SW1990 cells with IFI16 knockdown, and BMDM activation was completely blocked upon IFI16 knockdown (Figure 6I). Overexpression of IFI16 in SW1990 cells induced activation of co-cultured BMDMs in the absence of gemcitabine. Although induction of co-cultured BMDMs by IFI16-overexpressing SW1990 cells in the presence of gemcitabine may not be statistically significant (Figure 6J), which could be due to the high basic level, this observation in general consistently proved that IFI16 activation in PAAD cells could induce TAM activation in the tumor microenvironment.

## Discussion

Several studies have reported the role of TAMs in the tumorigenesis, development, and progression of PAAD (Krug et al., 2018; D’Errico et al., 2019; Zhang et al., 2019, 2020). TAMs promote PAAD growth by directly accelerating tumor cell expansion and spread (Ye et al., 2018) or indirectly generating the tumor-favoring immunosuppressive tumor microenvironment (Stromnes et al., 2019) or both (Mitchem et al., 2013). There are two subtypes of TAMs in the tumor microenvironment of PAAD: the pro-inflammatory M1 and immunosuppressive M2 phenotypes (Aras and Zaidi, 2017). It is far from conclusive which types of TAMs are predominant in mediating the tumor progression of PAAD, as studies have debated the pro-tumoral role of both TAMs (Partecke et al., 2013; Helm et al., 2014). Additionally, instead of the particular subtype of TAMs, the gross TAM amount was positively correlated with the poor prognosis of PAAD patients (Yu et al., 2019). Therefore, in our study, we studied the effect of depletion of the whole TAM population on IFI16-mediated tumor growth because the specific removal of either subtype of TAMs is perceptually and technically difficult. Removal of TAMs using liposome clodronate attenuated IFI16 overexpression-induced tumor growth in PAAD. IFI16-induced IL-1β expression is responsible for the induction of TAMs. Although IL-1β is generally considered a pro-inflammatory cytokine, we cannot directly conclude that the IL-1β-induced TAMs in our study were prone to pro-inflammatory M1 phenotypes. Indeed, IL-1β could initiate M2 macrophage polarization, which also contributes to tumor growth in head and neck squamous cell carcinoma (Chen et al., 2018). It is possible that the IFI16-overexpressing TAM population is a heterogenic population that contains the M1 and M2 phenotypes of macrophages, which both contribute to the tumor growth of PAAD.

In our study, we found that IL-1β plays an important role in regulating the tumor microenvironment of PAAD. Use of neutralizing antibodies against IL-1β can modulate the maturation, proliferation, and migration abilities of TAMs, which may retard tumor growth. However, our study does not exclude the possibility that secretion of IL-1β from PAAD cells, as observed in our study, has no autonomous action on tumor cells itself, which means that we did not completely rule out the possibility that IL-1β secretion can affect tumor cell expansion and spread. IL-1β secretion from TAMs was reported to promote the epithelial-to-mesenchymal transition of pancreatic tumor cells and therefore contribute to its distant metastasis (Chen et al., 2019). Additionally, IL-1β may also serve as a pro-tumoral factor by inducing cancer angiogenesis (Shchors et al., 2006) and cancer-associated fibroblasts (Brunetto et al., 2019). Further systematic investigation on the change in tumor and stromal cells upon IL-1β neutralization *in vivo* may be able to illustrate the overall effect of IL-1β on PAAD progression.

We also noticed that suppression of IFI16-induced inflammasome activation could significantly improve gemcitabine sensitivity. Gemcitabine remains the main treatment for patients with non-resectable PAAD; however, its efficacy in restricting tumor growth and prolonging patient survival is very limited, indicating the poor response of most PAAD to gemcitabine treatment. The reasons behind its poor responsiveness may be multiple, for instance, the primary resistance of PAAD cells due to the expression of multidrug resistance proteins and drug metabolism enzymes (Sarvepalli et al., 2019); however, we cannot rule out the possible involvement of the tumor microenvironment. Stromal cells, including TAMs, cancer-associated fibroblasts, myeloid-derived suppressor cells, and T lymphocytes, have been reported to be involved in gemcitabine resistance in PAAD (Thakur et al., 2013; Zhu et al., 2014; Wei et al., 2018; Halbrook et al., 2019). In our study, we found that gemcitabine treatment significantly induced the activation of the IFI16-related inflammasome and recruitment of TAMs. This further supports the role of TAMs in mediating gemcitabine sensitivity of PAAD tumors, although it is yet to be concluded that only TAMs play a dominant role. Gemcitabine could activate the tumor inflammasome, at least partially, by upregulating IFI16 expression. Gemcitabine is commonly reported as an inflammation inducer (Farr et al., 2017), which is considered an unfavorable factor when evaluating its treatment outcome in multiple types of cancers (Kim et al., 2020). Although we cannot fully conclude the mechanisms of IFI16 upregulation by gemcitabine treatment, it is postulated that gemcitabine, as a DNA damage agent, may cause the breakdown of dsDNA in both normal and cancer cells in the tumors (Jones et al., 2014). IFI16 was found to be a sensor of innate immunity in response to dsDNA (Morrone et al., 2014) and to mediate the activation of multiple cellular processes such as the inflammasome machinery. Further systemic investigation is required to determine whether dsDNA breakdown caused by gemcitabine majorly dominates the upregulation of IFI16 in cancer cells and reshapes the tumor microenvironment by increasing the TAM population in PAAD.

## Conclusion

In this study, we systematically examined the expression patterns and functional roles of inflammasome-related proteins in PAAD. We found that several proteins were upregulated in the PAAD tissues compared with those in the normal adjacent pancreas; however, only the upregulation of IFI16 correlated with the poor survival of PAAD patients. Overexpression of IFI16 significantly promoted the orthotopic growth of PAAD tumors in a murine model and altered the immune cell profile in the tumor microenvironment by increasing the TAM population. Moreover, depletion of TAMs attenuated IFI16-induced PAAD tumor growth. Overexpression of IFI16 in tumor cells activated inflammasome machinery, thereby inducing the production of IL-1β and causing maturation, proliferation, and migration of TAMs in the tumor microenvironment. Neutralization of IL-1β abolished the effect of IFI16-overexpressing tumor cells on TAMs. Additionally, knockdown of IFI16 in gemcitabine-treated PAAD tumors reduced TAM infiltration in the tumor microenvironment, improving gemcitabine sensitivity. Our study sheds light on the role of IFI16 as a potential target for the development of a novel therapeutic strategy for PAAD.

## Data Availability Statement

The raw data supporting the conclusions of this article will be made available by the authors, without undue reservation.

## Ethics Statement

The animal study was reviewed and approved by the Animal Experimental Ethics Committee of Ruijin Hospital, Shanghai Jiao Tong University School of Medicine.

## Author Contributions

JX and LZ conceived the idea, designed the experiments, analyzed the data, and wrote the manuscript. J-XC and C-SC conducted and wrote the experiments. H-FG and Z-JC conducted parts of the experiments. Z-JC, L-LL, J-YX, and X-HS revised the manuscript. All authors contributed to the article and approved the submitted version.

## Conflict of Interest

The authors declare that the research was conducted in the absence of any commercial or financial relationships that could be construed as a potential conflict of interest.

## Footnotes

1 http://gepia.cancer-pku.cn/

2 https://www.ncbi.nlm.nih.gov/geo
