# Supplementary material for: Overexpression of Interferon-Inducible Protein 16 Promotes Progression of Human Pancreatic Adenocarcinoma Through Interleukin-1β-Induced Tumor-Associated Macrophage Infiltration in the Tumor Microenvironment
Source: Front Cell Dev Biol. 2021 Jun 4;9:640786. doi: 10.3389/fcell.2021.640786 (PMC8213213; doi:10.3389/fcell.2021.640786)
Supplement: Supplementary file 1 [file Data_Sheet_1.DOCX]

**Supplemental materials**


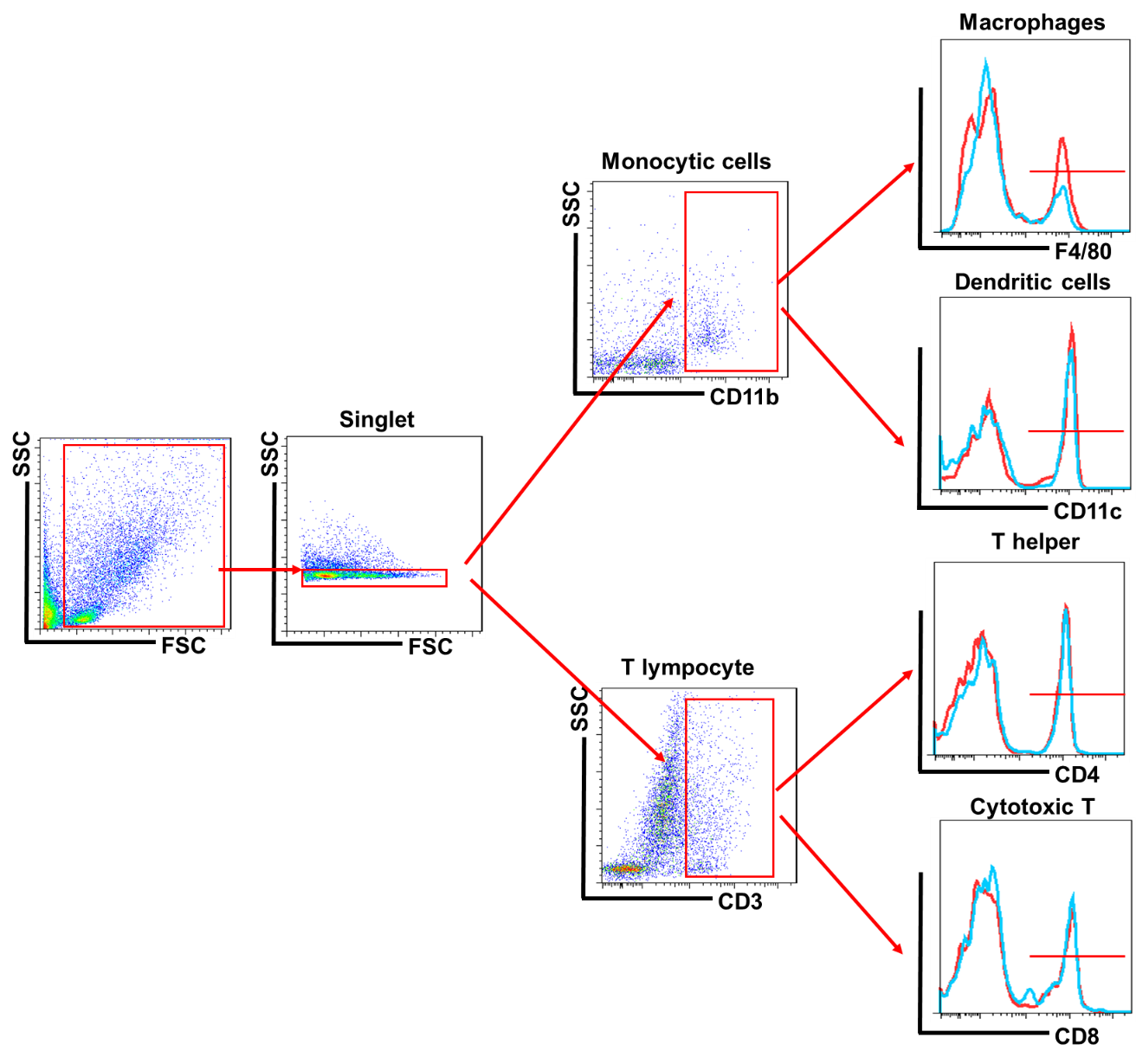


**Fig.S1 Gating strategy for identifying immune cells in the tumour microenvironment.**


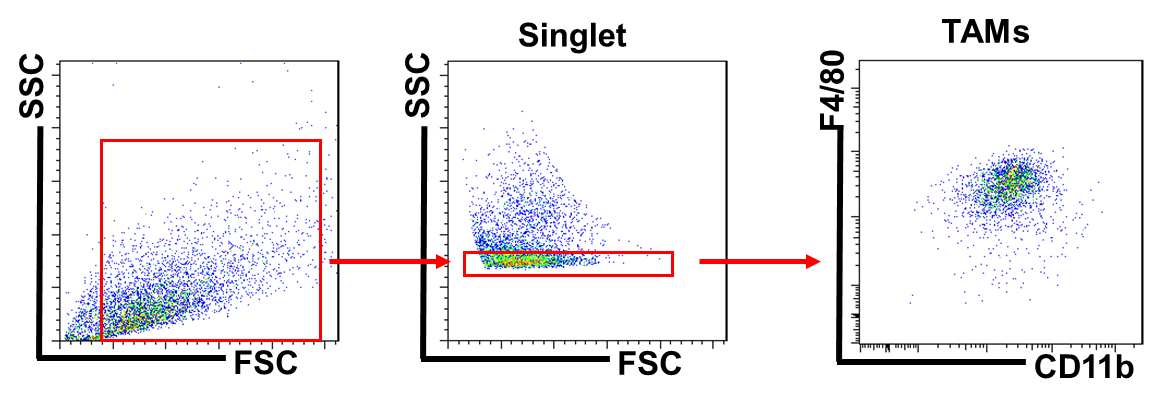


**Fig.S2 Gating strategy for in vitro culture of BMDMs with conditional medium from Panc-2 cells**


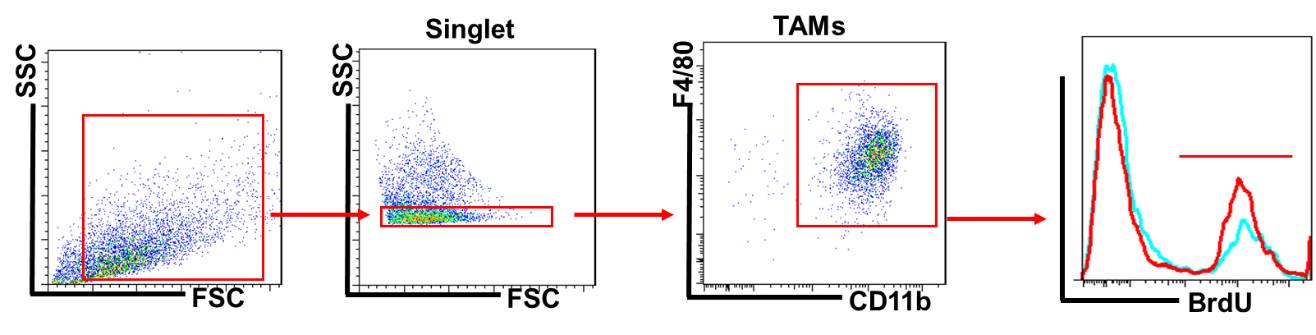


**Fig.S3 Gating strategy for in vitro BrdU incorporation assay**


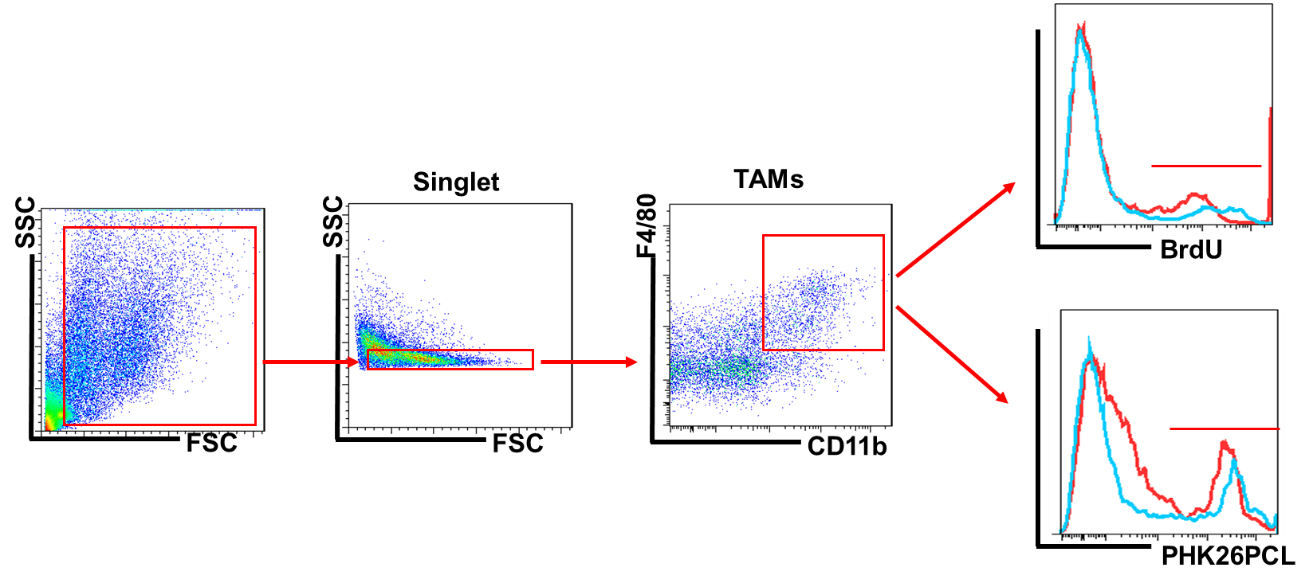


**Fig.S4 Gating strategy for in vivo BrdU incorporation assay and PKH26PCL staining**
